# System for the Recognizing of Pigmented Skin Lesions with Fusion and Analysis of Heterogeneous Data Based on a Multimodal Neural Network

**DOI:** 10.3390/cancers14071819

**Published:** 2022-04-03

**Authors:** Pavel Alekseevich Lyakhov, Ulyana Alekseevna Lyakhova, Nikolay Nikolaevich Nagornov

**Affiliations:** 1 North-Caucasus Center for Mathematical Research, North-Caucasus Federal University, 355017 Stavropol, Russia; ljahov@mail.ru; 2 Department of Automation and Control Processes, Saint Petersburg Electrotechnical University “LETI”, 197376 Saint Petersburg, Russia; sparta1392@mail.ru; 3 Department of Mathematical Modeling, North-Caucasus Federal University, 355017 Stavropol, Russia

**Keywords:** digital image processing, pattern recognition, convolutional neural networks, multimodal neural networks, heterogeneous data, metadata, dermatoscopic images, pigmented skin lesions, hair removal, melanoma

## Abstract

**Simple Summary:**

Skin cancer is one of the most common cancers in humans. This study aims to create a system for recognizing pigmented skin lesions by analyzing heterogeneous data based on a multimodal neural network. Fusing patient statistics and multidimensional visual data allows for finding additional links between dermoscopic images and medical diagnostic results, significantly improving neural network classification accuracy. The use by specialists of the proposed system of neural network recognition of pigmented skin lesions will enhance the efficiency of diagnosis compared to visual diagnostic methods.

**Abstract:**

Today, skin cancer is one of the most common malignant neoplasms in the human body. Diagnosis of pigmented lesions is challenging even for experienced dermatologists due to the wide range of morphological manifestations. Artificial intelligence technologies are capable of equaling and even surpassing the capabilities of a dermatologist in terms of efficiency. The main problem of implementing intellectual analysis systems is low accuracy. One of the possible ways to increase this indicator is using stages of preliminary processing of visual data and the use of heterogeneous data. The article proposes a multimodal neural network system for identifying pigmented skin lesions with a preliminary identification, and removing hair from dermatoscopic images. The novelty of the proposed system lies in the joint use of the stage of preliminary cleaning of hair structures and a multimodal neural network system for the analysis of heterogeneous data. The accuracy of pigmented skin lesions recognition in 10 diagnostically significant categories in the proposed system was 83.6%. The use of the proposed system by dermatologists as an auxiliary diagnostic method will minimize the impact of the human factor, assist in making medical decisions, and expand the possibilities of early detection of skin cancer.

## 1. Introduction

According to World Health Organization statistics, non-melanoma and melanoma skin cancer incidence has significantly increased over the past decade [1]. Up to three million cases of non-melanoma skin cancer [2] and about 140,000 cases of melanoma skin cancer are recorded annually [3]. According to the Skin Cancer Foundation Statistics [4], every third case of cancer diagnostics is caused by skin cancer, making it one of the most common types of malignant lesions in the body [5]. This is because the bulk of the population of the countries of the Northern Hemisphere of the Earth are owners of I and II skin phototypes according to Fitzpatrick’s classification [6]. A feature of these phototypes is the genetic inability to increase the level of Ultraviolet radiation (UV) [7] and the greatest tendency to develop melanoma [8]. In modern conditions of decreasing the thickness of the atmosphere’s ozone layer, UV directly affects the skin, a factor in the activation of oncogenes. It is estimated that a 10% decrease in the ozone layer will lead to an additional 300,000 non-melanoma and 4500 melanoma skin cancers [9]. In regions with high sun exposure, skin cancer is preceded by solar keratosis, the diagnosis of which can help prevent the transformation of pigmented skin lesions into a cancer-positive form [10].

Rapid and highly accurate early diagnosis of skin cancer can reduce patients’ risk of death [11]. When detected early, the 5-year survival rate for patients with melanoma is 99%. In the later stages of diagnosis, when the disease reaches the lymph nodes and metastasizes to distant organs, the survival rate in patients is only 27% [3]. Dermatoscopy is the most common method for diagnosing pigmented skin lesions visually [12]. This method is based on the visual acuity and experience of the practitioner and can only be effectively used by qualified professionals [13]. With the help of dermatoscopy, an experienced dermatologist can achieve an average accuracy in the classification of pigmented skin lesions that ranges from 65% to 75% [14]. The early manifestations of malignant and benign neoplasms are visually indistinguishable [15].

Today medicine is considered one of the strategic and promising areas for the effective implementation of systems based on artificial intelligence [16]. There is an improvement in mathematical models and methods, as well as an increase in the amount of digital information in various fields of medicine due to the accumulation of data from electronic medical records, the results of laboratory and instrumental studies, mobile devices for monitoring human physiological functions, etc. [17]. The development of artificial intelligence technologies allowed algorithms for computer analysis of data to be equal to inefficiency, and some tasks surpass human capabilities [18]. A comparison of the classification accuracy of pigmented skin lesions in dermatologists with different levels of experience and a computer program using an artificial intelligence algorithm is presented in articles such as [19,20,21]. Studies show that artificial intelligence can outperform 136 out of 157 dermatologists and achieve higher accuracy in recognizing pigmented lesions. Despite the higher quality of recognition in artificial intelligence systems than visual diagnostics in physicians, the problem of low accuracy in general in neural network classification systems remains relevant. One of the possible ways to improve recognition accuracy is using the image pre-processing stage [22].

There are many methods for pre-processing dermoscopic images to improve and visually highlight diagnostically significant features. One of these methods is segmentation to highlight pigmented skin lesions’ contours. Segmentation can be performed using a biorthogonal two-dimensional wavelet transform and the Otsu algorithm [23]. Edge extraction can be done using Gaussian contrast enhancement and edge extraction using the saliency map construction [24]. Saliency maps use inner and outer non-overlapping windows, making the foreground and background distinct. A significant disadvantage of segmentation methods using filters is the lack of versatility in selecting contours in images of different quality. Illumination, skin color, and sharpness of the contours of a pigmented skin lesion significantly reduce the accuracy of these algorithms. Another way to highlight contours on dermoscopic images is contrast stretching with further detection using Faster Region-Based Convolutional Neural Network (Faster R-CNN) [25,26]. Segmentation based on neural network algorithms makes it possible to accurately identify the contours of pigmented skin lesions, separate a pigmented neoplasm from a skin area, and exclude the influence of skin color type when recognized by artificial intelligence. At the same time, the problem of the presence of hair structures remains, which can be perceived by both neural network algorithms and filter-based algorithms as part of a pigmented skin lesion.

The presence of hair in dermatoscopic images can drastically change the size, shape, color, and texture of the lesion, which significantly affects the automatic analysis of the neural network [27]. Removing hair from images during digital pre-processing is an important step in improving the accuracy of automated diagnostic systems [28]. Today, several methods are designed for pre-processing dermatoscopic images of pigmented skin lesions to remove hair or other noise elements [29]. For example, the essence of the DullRazor process [30] is to use the morphological operation of closing. A significant drawback of DullRazor is the distortion of the dark areas of pigmented lesions, which can change diagnostic signs and have a substantial impact on the quality of recognition. In [31], another hair removal method on dermatoscopic images is presented based on non-linear Partial Differential Equation diffusion (PDE-diffusion). The algorithm is designed to fill linear hair structures by diffusion. This method is also used in [32,33].

Another way to improve the accuracy of intelligent classification systems is to combine heterogeneous data and further analyze them to find additional relationships. In database dermatology, heterogeneous data mining makes it possible to combine patient statistical metadata and dermoscopic images, greatly improving the recognition of pigmented skin lesions. The use of multimodal neural network systems [34,35,36,37], as well as methods for combining metadata and multidimensional visual data [38], has significantly improved the accuracy in recognizing pigmented skin lesions.

Despite significant progress in implementing artificial intelligence technologies to analyze dermatological data, developing neural network systems of varying complexity is relevant to achieving higher recognition accuracy. The main hypothesis of the manuscript is a potential increase in the quality of neural network systems for analyzing medical data due to the emerging synergy when using various methods to improve recognition accuracy together. This study aims to develop and model a multimodal neural network system for analyzing dermatological data through the preliminary cleaning of hair structures from images. The proposed system makes it possible to achieve higher recognition accuracy levels than similar neural network systems due to the preliminary cleaning of hair structures from dermoscopic images. The use of the proposed system by dermatologists as an auxiliary diagnostic method will minimize the impact of the human factor in making medical decisions.

The rest of the work is structured as follows. Section 2 is divided into several sub-section. In Section 2.1 a description of a method for identifying and cleaning hair structures as pre-processing dermatoscopic images of pigmented skin lesions is proposed. In Section 2.2 a description of the method for pre-processing statistical metadata about patients has been made. In Section 2.3 the definition of a multimodal neural network system for processing statistical data and dermatoscopic images of pigmented skin lesions is presented. Section 3 presents practical modeling of the proposed multimodal neural network system to classify pigmentary neoplasms with a preliminary stage of hair removal on dermatoscopic images. Section 4 discusses the results obtained and their comparison with known works in neural network classification of dermatoscopic skin images. In conclusion, the results of the work are summed up.

## 2. Materials and Methods

**Figure 1:**
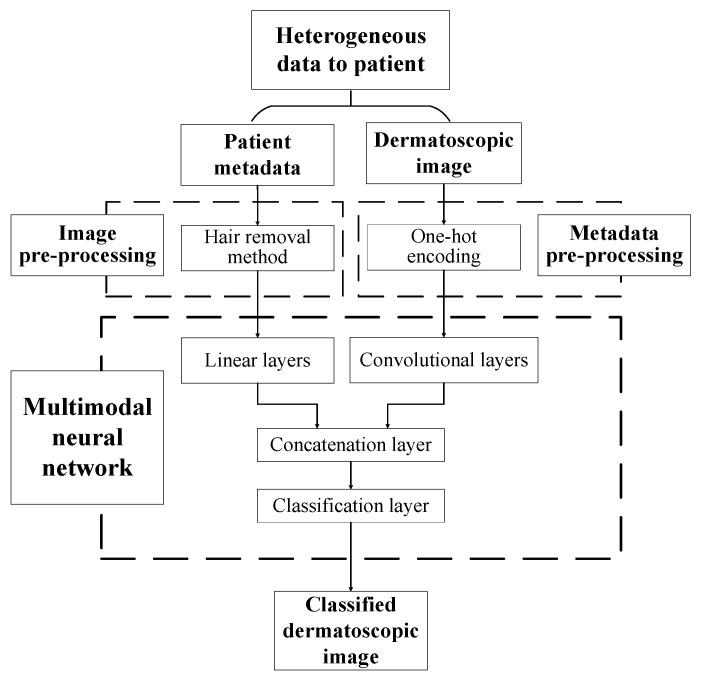
Multimodal neural network system for the classification of dermatoscopic images of pigmented skin lesions with preliminary heterogeneous data processing.

The paper proposes a multimodal neural network system for recognizing pigmented skin lesions with a stage of preliminary processing of dermatoscopic images. The proposed multimodal neural network system for analysis and classification combines heterogeneous diagnostic data represented by multivariate visual data and patient statistics. The scheme of a multimodal neural network system for the classification of dermatoscopic images of pigmented skin lesions with preliminary processing of heterogeneous data is shown in Figure 1.

The multidimensional visual data undergoes a pre-processing stage, which identifies and cleans hair structures from dermatoscopic images of pigmented skin lesions. Patient statistics also undergo a one-hot encoding process to generate a feature vector. The multimodal neural network system for recognizing pigmented lesions in the skin consists of two neural network architectures. Dermatoscopic images are processed using the specified Convolutional Neural Network (CNN) architecture. Statistical metadata is processed using a linear multilayer neural network. The resulting feature vector at the CNN output and the output signal of the linear neural network are combined on the concatenation layer. The combined signal is fed to the layer for classification. The output signal from the proposed multimodal neural network system for recognizing pigmented skin lesions is the percentage of 10 diagnostically significant categories, including a recognized dermatoscopic image.

### 2.1. Hair Removal

The main diagnostic method in the field of dermatology is visual analysis. Today, many imaging approaches have been developed to help dermatologists overcome the problems caused by the apperception of tiny skin lesions. The most widely used imaging technique in dermatology is dermatoscopy, a non-invasive technique for imaging the skin surface using a light magnifying device and immersion fluid [39]. Statistics show that dermatoscopy has increased the efficiency of diagnosing malignant neoplasms by 50% [40]. A significant problem when working with this method is the possible presence of hair on the area of the pigmented lesion, which causes occlusion.

**Figure 2:**
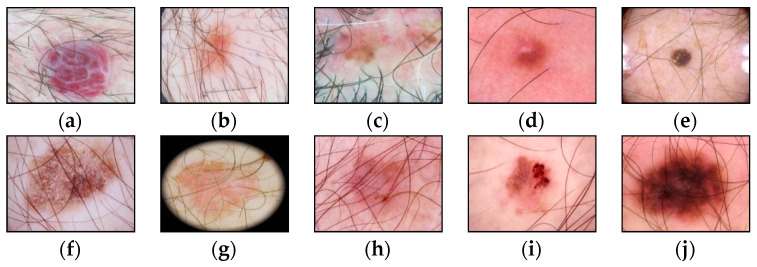
Examples of pigmented skin lesions images with hairy structures: (**a**) vascular lesions; (**b**) nevus; (**c**) solar lentigo; (**d**) dermatofibroma; (**e**) seborrheic keratosis; (**f**) benign keratosis; (**g**) actinic keratosis; (**h**) basal cell carcinoma; (**i**) squamous cell carcinoma; (**j**) melanoma.

The presence of such noisy structures as hair significantly complicates the work of dermatologists and specialists. It can also cause errors in recognizing pigmented skin lesions in automatic analysis systems. Hair violates the geometric properties of the pigmented lesion areas, which negatively affects the diagnostic accuracy [41]. Figure 2 shows dermatoscopic images of pigmented skin lesions with hair structures present that cause occlusion by altering the size, shape of the lesion, and texture of the image.

The most common way to solve the occlusion problem of pigmented skin lesions is to remove the visible part of the hair with a cutting instrument before performing a dermatoscopic examination. However, this approach leads to skin irritation. Also, it causes diffuse changes in the color of the entire pigmented lesion, which distorts diagnostically significant signs to a greater extent than the presence of hair itself. An alternative solution is digitalizing dermatoscopic visual data to remove hair structures. The essence of the hair pre-cleaning methods is to identify each pixel of the image as a pixel-hair or pixel-skin and then replace the pixels of the hair structures with skin pixels [42]. Preliminary digital processing of dermatoscopic images using morphological operations is one of the possible methods for identifying and replacing pixels of hair structures.

**Figure 3:**
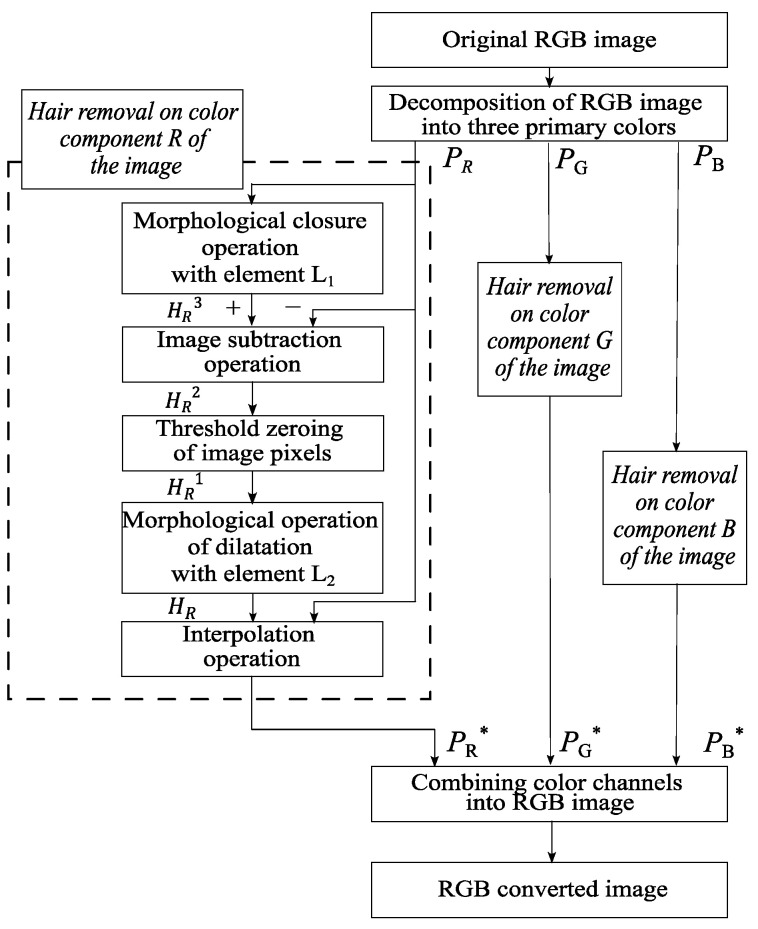
Scheme of the proposed method of identification and hair removal from dermatoscopic images of pigmented skin lesions.

This paper proposes a method for digital pre-processing dermoscopic images using morphological operations on multidimensional visual data. A step-by-step scheme of the proposed method is shown in Figure 3.

Image processing of pigmented skin lesions consists of four main stages. At the first stage, the RGB image is decomposed into color components. The second step is to locate the locations of the hair structures. At the third stage, the hair pixels are replaced with neighboring pixels. The fourth step is to reverse engineer an RGB color dermatoscopic image.

The input of the proposed method is RGB dermatoscopic images of pigmented neoplasms of the skin $P_{\left(x,y\right)}$. The color components $P_{R}$, $P_{G},$ and $P_{B}$ are extracted from the image. The following processing steps are performed separately for each color component. The variables $L_{1}$ and $L_{2}$ are defined as follows:

(1) $$L_{1,2}=\left\{\left(x,y\right):\rho \left(T,\left(x,y\right)\right)\leq r\right\}$$

where ρ is the distance from the center T of the set $L_{1,2}$ by the chosen metric, and r is the radius of the set specified by the user. The next stage is a morphological closure operation using the $L_{1}$ element to determine the location of hair structures on dermatoscopic images:

(2) $$H_{CC}{}^{3}=P_{CC}\;\cdot \;L_{1}=\left(P_{CC}⊕\;L_{1}\right)⊖\;L_{1}$$

where CC stands for the color channel, CC∈{R, G,B}, ⊕ is the operation of dilatation of the set P along $L_{1}$ and ⊖ is the operation erosion by element $L_{1}$. The closure operation smooths out the contours of the hair structures in dermatoscopic images, eliminates voids, and fills in narrow gaps and long small-width depressions.

At the next stage, the original image $P_{CC}$ is subtracted from the image obtained as a result of the $H_{CC}{}^{3}$ close operation:

(3) $$H_{CC}{}^{2}=H_{CC}{}^{3}-P_{CC}$$

The operator of zeroing the pixels δ of the image $P_{\left(x,y\right)}$ for further operations is defined as follows:

(4) $$\delta \left(P_{\left(x,y\right)}\right)=\{\begin{array}{c} P_{\left(x,y\right)},\;\mathrm{if}\;P_{\left(x,y\right)}>K\\ 0,\mathrm{if}\;P_{\left(x,y\right)}\leq K\; \end{array}$$

where K is the user-defined threshold of pixel intensity values. The next stage is the threshold zeroing of the pixels of the detected hair structures. For this, the entered zeroing operator δ is applied to the resulting dermatoscopic image $H_{CC}{}^{2}$:

(5) $$H_{CC}{}^{1}=\delta (H_{CC}{}^{2})$$

After the operation of threshold zeroing of pixels, a morphological operation of dilatation with the $L_{2}$ element is performed to expand the boundaries of the hair structures:

(6) $$H_{CC}=H_{CC}{}^{1}⊕\;L_{2}$$

The next step is to replace the pixels of the hair structure with neighboring pixels. Using the Laplace equation, pixels are interpolated from the area’s border of the selected hair structures. In this case, the pixels from the border of the hair structures cannot be changed. The last step is the reverse construction of the RGB color image from the extracted color components. For this, the color channels $P_{R}{}^{*}$, $P_{G}{}^{*}$, and $P_{B}{}^{*}$ are combined.

**Figure 4:**
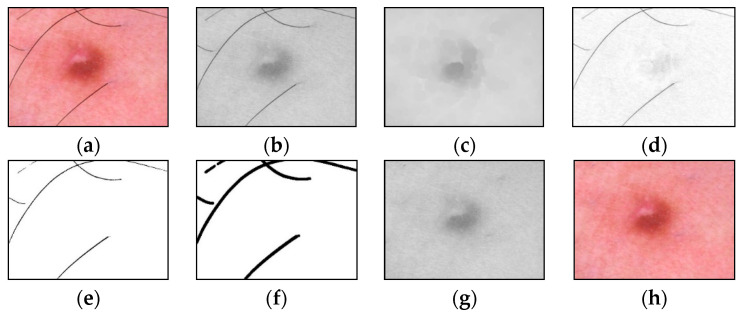
Images obtained as a result of passing each stage of the method of identification and hair removal: **(a**) input RGB image $P_{RGB}$; (**b**) the color component $P_{R}$, presented in shades of gray; (**c**) the result of the $H_{R}{}^{3}$ closing operation; (**d**) the result of the subtraction operation $H_{R}{}^{2}$ (inverted image); (**e**) the result of zeroing pixels $H_{R}{}^{1}$ (inverted image); (**f**) the result of the $H_{R}$ dilatation operation (inverted image); (**g**) pixel interpolation result $P_{R}{}^{*}$; (**h**) output RGB image $P_{RGB}{}^{*}$. Scale bar or magnification.

An example of the step-by-step work of the proposed method for identifying and cleaning hair structures from dermatoscopic images of pigmented skin lesions is shown in Figure 4. To improve the visual perception of the intermediate results of each method stage, Figure 4d–f were inverted.

### 2.2. Metadata Pre-Processing

Today, in medicine, there is an increase in the volume of digital information due to the accumulation of data from electronic medical records, the results of laboratory and instrumental studies, mobile devices for monitoring human physiological functions, and others [17]. Patient biomedical statistics are structured data that describe the characteristics of research subjects. Statistical data includes gender, age, race, predisposition to various diseases, bad habits, etc. Such information facilitates the search for connections between research objects and the analysis result.

Metadata pre-processing is converting statistical data into the format required by the selected data mining method. Since the proposed multimodal system for recognizing pigmented skin lesions is a fully connected neural network, it must encode the data as a vector of features. A corresponding metadata information vector is generated for each image in the dataset, which depends on the amount and type of statistical information. One-hot encoding can sometimes outperform complex encoding systems [43]. All multi-categorical variables (discrete variables with more than two categories) are converted to a new set of binary variables for one-hot encoding. For example, the categorical variable to denote a pigmented lesion on the patient’s body will be replaced by 8 dummy variables indicating whether the pigmented lesion is located on the anterior torso, head/neck, lateral torso, lower extremity, oral/genital, palms/soles, posterior torso, or upper extremity.

Suppose the M metadata includes various statistics $M=\left\{M_{1},\;M_{2},\;\ldots \;,\;M_{n}\right\}$ with $M_{n}\in m_{n}$, where $m_{n}$ is a pointer to a specific patient parameter. If $m_{n}$ is a pointer to the gender of the patient, then $M_{1}=\left\{male,\;female\right\}$. For each set $M_{n}$, which is one of the patient’s indicators, its power $\mu_{n}=\left|M_{n}\right|\;$is calculated. For metadata pre-processing, an $\vec{m}$ feature vector of $\sum_{n}\mu_{n}$ the dimension is generated. The first coordinate of the $\vec{m}$ metadata vector of the $\mu_{1}$ the dimension will encode the statistical data $m_{1}$. The next coordinate of the $\mu_{2}$ the dimension will encode the $m_{2}$ statistical data, and so on.

**Figure 5:**
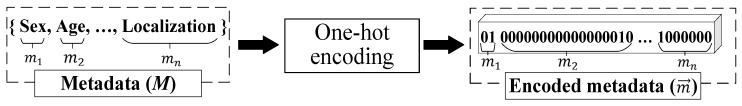
Metadata pre-processing scheme.

One-hot encoding is used to encode the statistic $m_{n}\in M_{n}$ as follows. For the set of $M_{n}$, the ordering is performed in an arbitrary fixed way for all considered cases. After that, the binary code $\underset{\mu_{n}}{\underbrace{1000\ldots 0}}\;$is reserved for the first element of the set $M_{n}$. For the second element of the set $M_{n}$, the binary code $\underset{\mu_{n}}{\underbrace{0100\ldots 0}}\;$is reserved, and so on. The statistical metadata pre-processing scheme is shown in Figure 5.

### 2.3. Multimodal Neural Network

In deep learning, multimodal fusion or heterogeneous synthesis combines different data types obtained from various sources [44]. In the field of diagnosis of pigmented skin lesions, the most common types of data are dermatoscopic images and patient statistics such as age, sex, and location of the pigmented lesion on the patient’s body. Combining visual data, signals, and multidimensional statistical data about patients allows you to create heterogeneous medical information databases that can be used to build intelligent systems for diagnostics and decision support for specialists, doctors, and clinicians [45]. The rationale for using heterogeneous databases is that the fusion of heterogeneous data can provide additional information and increase the efficiency of neural network analysis and classification systems [46]. The use of heterogeneous data in training multimodal neural network systems will improve the accuracy of diagnostics by searching for connections between visual objects of research and statistical metadata [47].

For the recognition of multidimensional visual data, the most optimal neural network architecture is CNN [48]. The input of the proposed multimodal system for neural network classification of pigmented skin lesions is supplied with dermatoscopic images of $P_{\left(img\right)}$, pre-processed metadata in the vector form of $\vec{m}=\left(m_{1},m_{2},\ldots ,m_{n}\right)\;$and tags with a diagnosis of $l\in \left\{1,\ldots ,N_{lab}\right\}$, where $N_{lab}$ is the number of diagnostic categories.

The dermatoscopic image includes R rows, C columns, and D color components. In this case, for the RGB format =3, the color components are represented by the levels of red, green, and blue colors of the image pixels. The input of the convolutional layer receives a dermatoscopic $P_{\left(img\right)}$ image, while the input is a three-dimensional function P(x,y,z), where 0≤x<R, 0≤y<C and 0≤z<D are spatial coordinates, and the amplitude P at any point with coordinates (x, y, z) is the intensity of the pixels at a given point. Then the procedure for obtaining feature maps in the convolutional layer is as follows:

(7) $$P_{f}\left(x,y\right)=g+\sum_{i=\frac{-w-1}{2}}^{\frac{w-1}{2}}\sum_{j=\frac{-w-1}{2}}^{\frac{w-1}{2}}\sum_{k=0}^{D-1}w_{ijk}^{\left(1\right)}P\left(x+i,\;y+j,k\right),$$

where $P_{f}$ is a feature map; $w_{ijk}^{\left(1\right)}$ is the coefficient of a filter of size w×w for processing D arrays; g is offset.

The concatenation layer at the input receives the feature map, which was obtained on the last layer intended for processing dermatoscopic images $P_{f}$, and the metadata vector $\vec{m}$. The $P_{f}$ feature map contains a set of $x_{ijk}$, where i is the height coordinate, j is the width coordinate, k is the number of the map obtained on the last layer from the set of layers that were intended for processing dermatoscopic images. The operation of combining heterogeneous data on the concatenation layer can be represented as follows:

(8) $$f_{l}=\sum_{i}\sum_{j}\sum_{k}x_{ijk}w_{ijkl}^{\left(2\right)}+\sum_{i=1}^{n}m_{i}w_{il}^{\left(3\right)},$$

where $w_{ijkl}^{\left(2\right)}$ is a set of weights for processing feature maps of dermatoscopic images; $w_{il}^{\left(3\right)}$ is a set of weights for processing metadata vectors.

**Figure 6:**
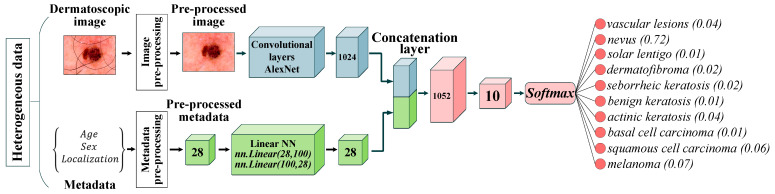
Neural network architecture for multimodal classification of pigmented skin lesions based on CNN AlexNet. Scale bar or magnification.

The activation of the last layer of the multimodal neural network is displayed through the softmax function with the distribution P(y|x, θ) and has the form:

(9) $$P\left(y|x,\;\theta \right)=softmax\left(x;\theta \right)=\frac{\exp{\left(w_{l}^{n}\right)}^{T}x_{l}^{n}+g_{l}^{n}}{\sum_{k=1}^{K}\exp{\left(w_{l}^{n}\right)}^{T}x_{l}^{n}+g_{l}^{n}}\;,$$

where $w_{l}^{n}$ is the weight vector leading to the output node that is associated with class l. The proposed multimodal system for recognizing pigmented skin lesions based on CNN AlexNet is shown in Figure 6.

## 3. Results

**Figure 7:**
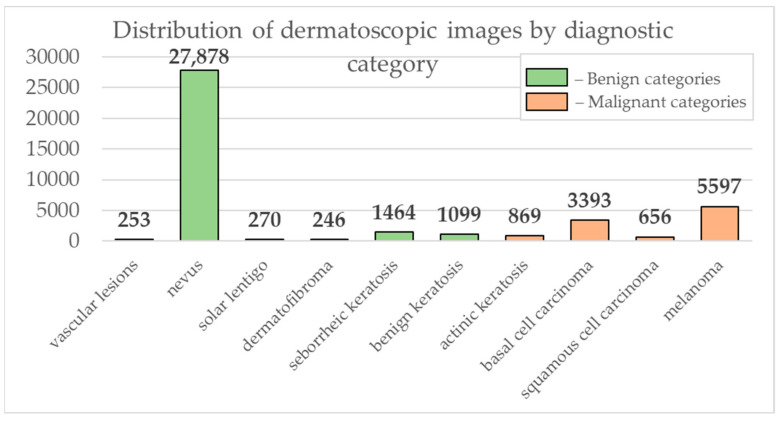
Diagram of the distribution of the number of dermatoscopic images in 10 diagnostically significant categories.

**Figure 8:**
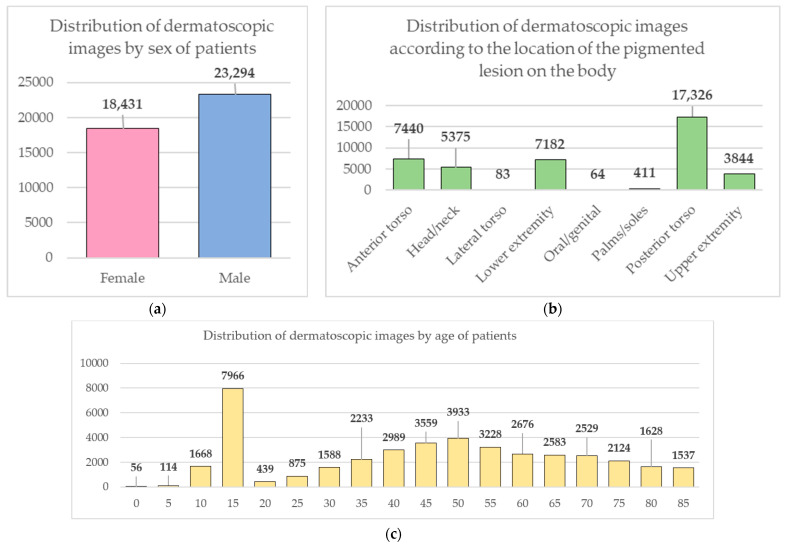
Diagrams of the distribution of the base of dermatoscopic images according to the statistical data of patients: (**a**) by gender; (**b**) by age; (**c**) by the location of the pigmented lesion on the body.

Data from the open archive of The International Skin Imaging Collaboration (ISIC), which is the largest available set of confidential data in dermatology, was used for the simulations [49]. The main clinical goal of the ISIC project is to support efforts to reduce mortality associated with melanoma and reduce biopsies by improving the accuracy and efficiency of early detection of melanoma. ISIC develops proposed digital imaging standards and engages the dermatological and bioinformatics communities to improve diagnostic accuracy using artificial intelligence. While the initial focus in the ISIC collaboration is on melanoma, diagnosing non-melanoma skin cancer and inflammatory dermatoses is equally important. ISIC has developed an open-source platform for hosting images of skin lesions under Creative Commons licenses. Dermatoscopic photos are associated with reliable diagnoses and other clinical metadata and are available for public use. The ISIC archive contains 41,725 dermatoscopic photographs of various sizes, representing a database of digital representative images of the 10 most important diagnostic categories. Most of the photographs are digitized transparencies of the Roffendal Skin Cancer Clinic in Queensland, Australia, and the Department of Dermatology at the Medical University of Vienna, Austria [50]. The dataset also contains statistical meta-information about the patient’s age group (in five-year increments), anatomical site (eight possible sites), and gender (male/female). Figure 7 shows a diagram of the distribution of dermatoscopic images for 10 diagnostically significant categories. Diagnostically significant categories are divided into groups “benign” and “malignant”, and are also arranged in order of increasing risk and severity of the course of the disease. Since actinic keratosis can be considered as intraepithelial dysplasia of keratinocytes and, therefore, as a “precancerous” skin lesion, or as in situ squamous cell carcinoma, this category was therefore assigned to the group of “malignant” pigmented neoplasms [51,52,53]. The diagram shows how unbalanced the available images of pigmented skin lesions are towards the “nevus” category. Figure 8 shows diagrams of the distribution of the base of dermatoscopic images according to the statistical data of patients. The database is dominated by male patients and patients aged 15 to 20 years. At the same time, in patients, pigmented skin lesions were most often found on the back (posterior torso).

The modeling was performed using the high-level programming language Python 3.8.8. All calculations were performed on a PC with an Intel (R) Core (TM) i5-8500 CPU @ 3.00 GHz 3.00 GHz with 16 GB of RAM and a 64-bit Windows 10 operating system. Multimodal CNN training was carried out using a graphics processing unit (GPU) based on an NVIDIA video chipset GeForce GTX 1050TI.

**Figure 9:**
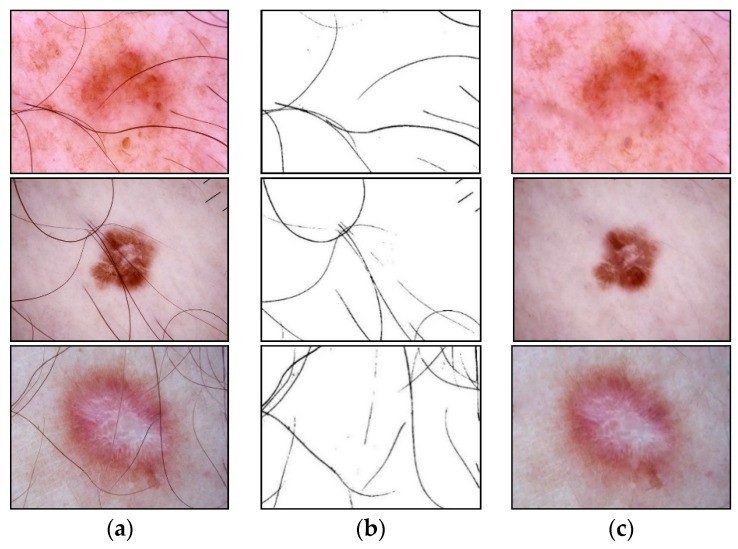
Examples of identification and cleaning of hair structures from dermatoscopic images of pigmented skin lesions using the proposed method: (**a**) original dermatoscopic image; (**b**) the result of extracting hair in the image (inverted image); (**c**) dermatoscopic image cleared of hair structures. Scale bar or magnification.

Preliminary heterogeneous data processing was carried out at the first stage of the proposed multimodal classification system. Dermatoscopic image pre-processing consisted of stepwise hair removal and image resizing. The removal of hair structures was carried out using the developed method based on morphological operations, presented in Section 2.1. An empirical analysis of the application of Formula (1) showed that the best result of identification and cleaning of hair structures is achieved at r=5 for the element $L_{1}$ and at r=3 for the element $L_{2}$. In the calculations, the Euclidean norm (L2) was used as a metric. It was also empirically found that the optimal threshold value in Formula (4) is K=40. Examples of pre-cleaning dermatoscopic images are shown in Figure 9. Figure 9b was inverted to improve the visual perception of the results of the stage of hair extraction in the pictures.

**Figure 10:**
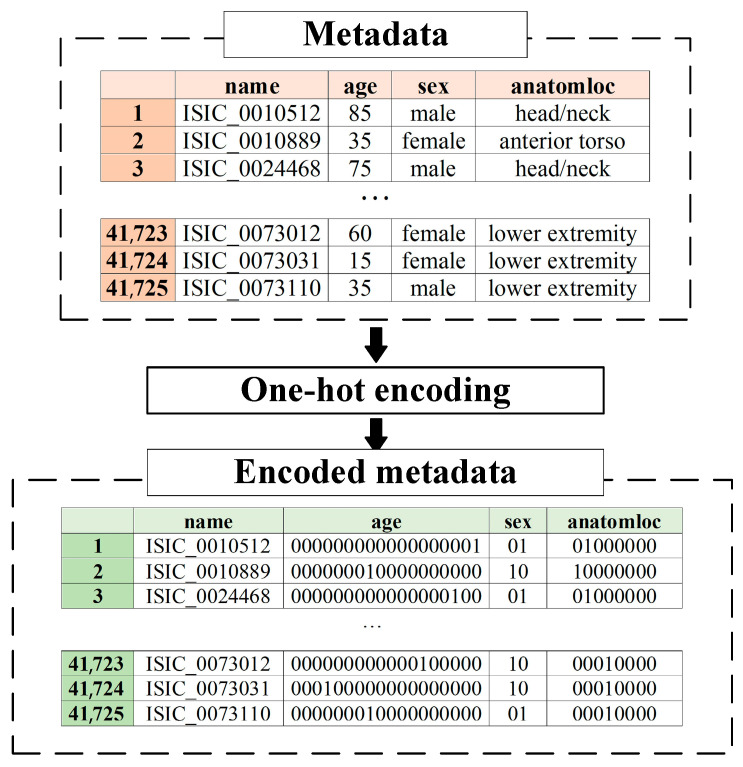
An example of pre-processing statistical patient metadata using one-hot encoding.

The pre-processing of patient metadata consisted of one-hot encoding to convert the vector format required for further mining. The coding tables for each patient metadata index are presented in Table 1, Table 2 and Table 3. An example of pre-processing statistical patient metadata using one-hot encoding is shown in Figure 10. 

CNN AlexNet [54], SqueezeNet [55], and ResNet-101 [56] were selected to simulate a multimodal neural network system for recognizing pigmented skin lesions, which were pre-trained on a set on a set of natural images ImageNet. The most common size of dermatoscopic images in the ISIC database is 450×600×3, where 3 is the color channels. For neural network architectures AlexNet and SqueezeNet, the images were transformed to a size of 227×227×3. For CNN ResNet-101, the images were converted to 224×224×3. For further modeling, the base of dermatoscopic photographs was divided into images for training and images for validation in a percentage ratio of 80 to 20. Since the ISIC dermatoscopic image base is strongly unbalanced towards the “nevus” category, the training images were augmented using affine transformations.

Large volumes of training data make it possible to increase the classification accuracy of automated systems for neural network recognition of dermatoscopic images of pigmented skin lesions. Creating large-scale medical imaging datasets is costly and time-consuming because diagnosis and further labeling require specialized equipment and trained practitioners. It also requires the consent of patients to process and provides personal data. Existing training datasets for the intelligent analysis of pigmented skin lesions, including the ISIC open archive, are imbalanced across benign lesion classes. All of this leads to inaccurate classification results due to CNN overfitting.

Affine transformations are one of the main methods for increasing and balancing the amount of multidimensional visual data in each class. The possible affine transformations are rotation, displacement, reflection, scaling, etc. The selected dermatoscopic images of pigmented skin lesions include multidimensional visual data of various sizes. Different CNN architectures require input images of a certain size. Scaling using affine transformations transforms visual data into a set of images of the same size. Scaling is usually combined with cropping to achieve the desired image size.

**Figure 11:**
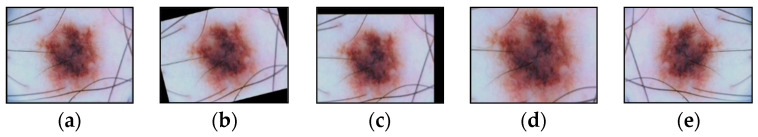
Images obtained as a result of affine transformations: (**a**) original image; (**b**) image after the operation of rotation by a given angle; (**c**) image after shift operation; (**d**) image after the scaling operation; (**e**) image after the reflection operation. Scale bar or magnification.

Augmentation of dermatoscopic images of pigmented skin lesions included all of the above methods of affinity transformations, examples of which are shown in Figure 11.

**Figure 12:**
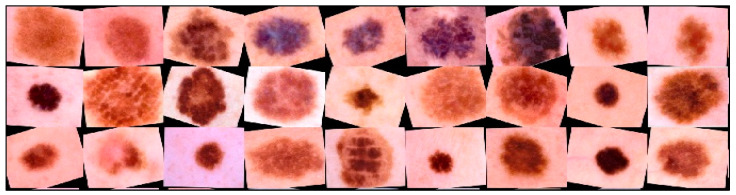
Examples of dermatoscopic training images that have been previously cleaned and enlarged using affinity transformations. Scale bar or magnification.

New multidimensional visual data were created from existing ones using augmentation for more effective training. This allowed us to increase the number of training images. Training data augmentation has proven effective enough to improve accuracy in neural network recognition systems for medical data [57]. When trained, neural network classifiers tend to lean towards classes containing the largest number of images [58]. The use of data augmentation made it possible to minimize the imbalance and achieve uniform learning across all diagnostically significant classes presented. An example of transformed dermatoscopic images from the database for training a multimodal neural network for recognizing pigmented skin lesions is shown in Figure 12.

**Figure A1:**
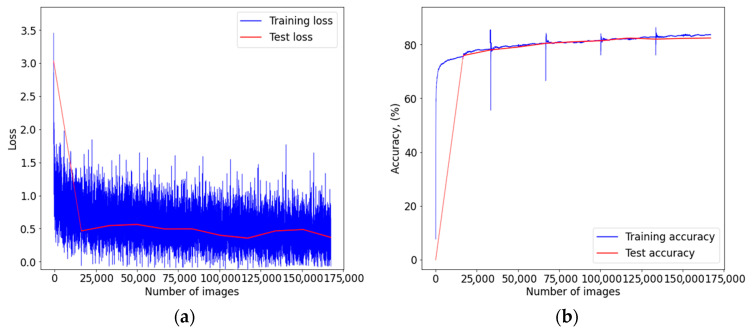
Graph of learning outcomes of a multimodal neural network system for classifying dermatoscopic images of pigmented skin lesions based on CNN AlexNet: (**a**) loss function; (**b**) recognition accuracy.

**Figure A2:**
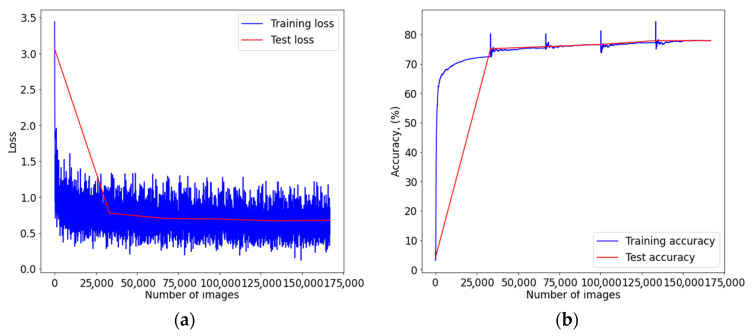
Graph of learning outcomes of a multimodal neural network system for classifying dermatoscopic images of pigmented skin lesions based on CNN SqueezeNet: (**a**) loss function; (**b**) recognition accuracy.

**Figure A3:**
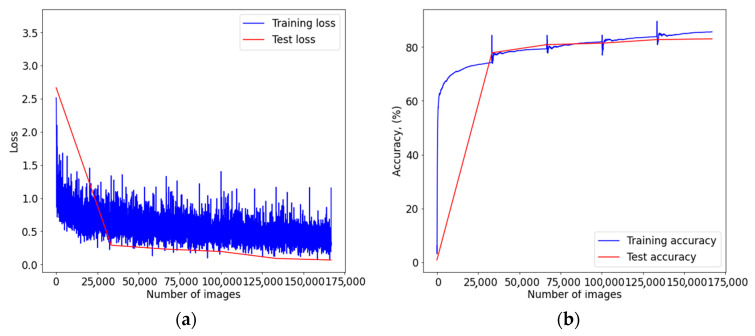
Graph of learning outcomes of a multimodal neural network system for classifying dermatoscopic images of pigmented skin lesions based on CNN ResNet-101: (**a**) loss function; (**b**) recognition accuracy.

Pre-processed images of pigmented skin lesions were fed into CNN architectures. The vector of pre-processed metadata was provided to the input of a linear neural network, which consisted of several linear layers and ReLu activation layers. After passing the different input signals through the CNN and the linear neural network, the heterogeneous data passed fusion on the concatenation layer. The combined data was fed to the softmax layer for classification. Figure A1, Figure A2 and Figure A3 from Appendix A show graphs of the learning outcomes of a multimodal neural network system for recognizing pigmented skin lesions based on various CNNs.

Table 4 presents the results of assessing the recognition accuracy of dermatoscopic images of pigmented skin lesions. The highest indicator of the accuracy of recognition of pigmented skin lesions was achieved using a multimodal neural network system for recognizing pigmented skin lesions with a stage of preliminary hair cleaning with a pre-trained AlexNet architecture [54] and amounted to 83.56%. When training each multimodal neural network architecture using the method of preliminary identification and cleaning of hair structures, the obtained percentage of recognition accuracy was higher than when training original CNNs without a preliminary processing stage. The increase in recognition accuracy during training of multimodal neural network recognition systems for pigmented skin lesions with a stage of preliminary hair cleaning was 4.93–6.28%, depending on the CNN architecture. The best indicator of improving the recognition accuracy was obtained when training a multimodal neural network classification system with a preliminary hair cleaning stage with a pre-trained ResNet-101 [56] architecture amounted to 6.28%. The smallest result of an increase in recognition accuracy of 4.93% was shown by a multimodal system based on AlexNet [54]. Adding each of the components to the system improves the accuracy by 2.18–4.11%. As a result of modeling the original CNN architecture with the stage of preliminary cleaning of hair structures based on SqueezeNet, the increase in recognition accuracy was 2.13%. At the same time, adding the stage of neural network analysis of statistical data made it possible to increase the accuracy by another 4.11%. For the AlexNet neural network architecture, this increase was 2.18% and 2.75%, respectively. For the ResNet-101 neural network architecture, recognition accuracy increased by 3.17% and 3.11%, respectively. The results obtained indicate that the combined use of various methods for improving the accuracy of recognition can significantly increase the accuracy of neural network data analysis.

The results predicted by the multimodal neural network from the test sample were converted to a binary form to construct the Receiver Operating Characteristic curve (ROC curve). Each predicted class label consisted of a combination of two characters with a length of 10 characters. The ROC curve represents the number of correctly classified positive values on incorrectly classified negative values.

(10) $$TPR=\frac{TP}{TP+FN}\times 100\%$$

(11) $$FPR=\frac{FP}{TN+FP}\times 100\%,$$

where TP is true positive cases; TN is true negative cases; FN is false-negative cases; FP is false positives cases. The ROC curve is plotted so that the x-axis is the proportion of false positives FPR, and the y-axis is the proportion of true positive TPR cases. The AUC is the area under the ROC curve and is calculated as follows:

(12) $$AUC=\int_{0}^{1}TPR\;d\left(FPR\right).$$

**Figure 13:**
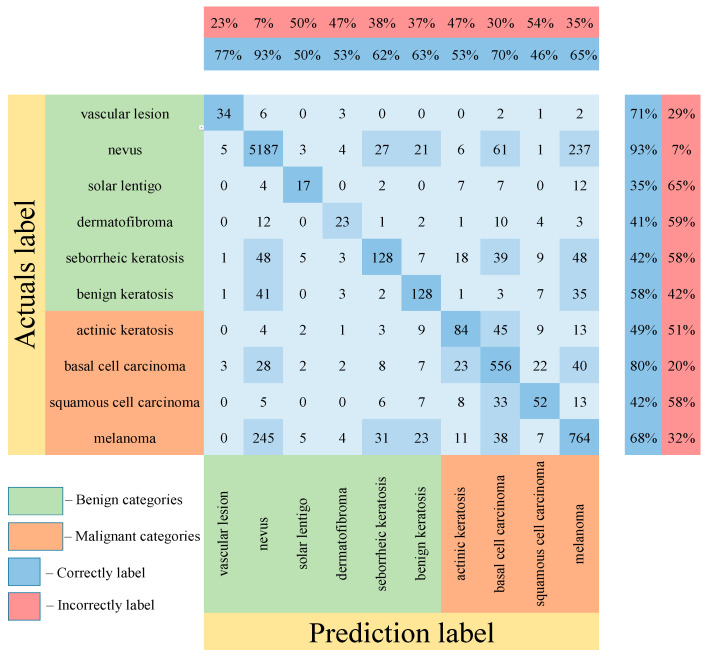
Confusion matrix in the testing results in a multimodal neural network system for recognizing pigmented skin lesions based on CNN AlexNet.

**Figure 14:**
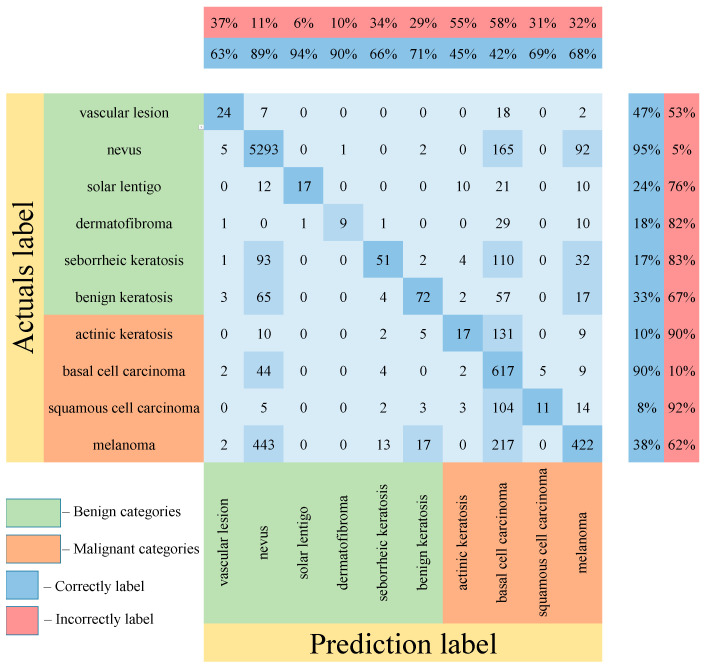
Confusion matrix in the testing results in a multimodal neural network system for recognizing pigmented skin lesions based on CNN SqueezeNet.

**Figure 15:**
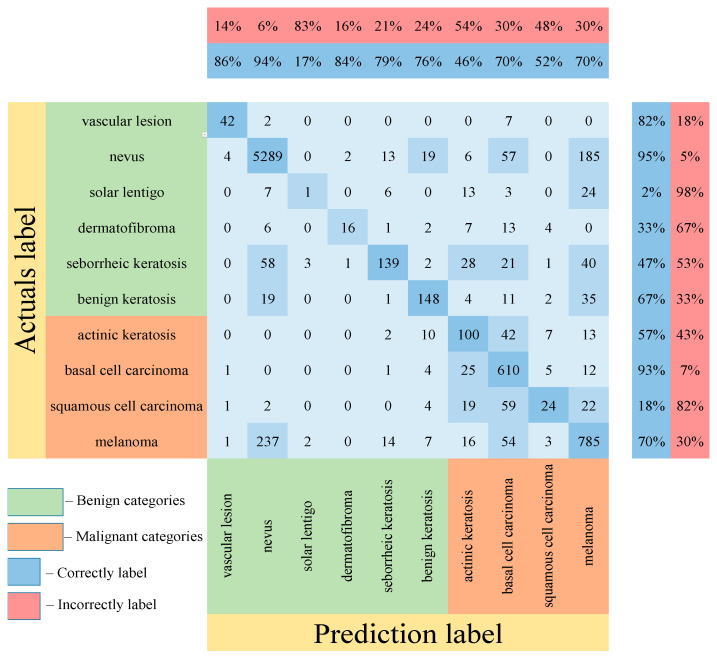
Confusion matrix in the testing results in a multimodal neural network system for recognizing pigmented skin lesions based on CNN ResNet-101.

Table 5 shows the results of testing the proposed multimodal neural network system for recognizing pigmented lesions with a stage of preliminary cleaning from hair structures. Figure 13, Figure 14 and Figure 15 show confusion matrices resulting from testing multimodal neural network systems for identifying pigmented skin lesions based on various CNNs.

**Figure 16:**
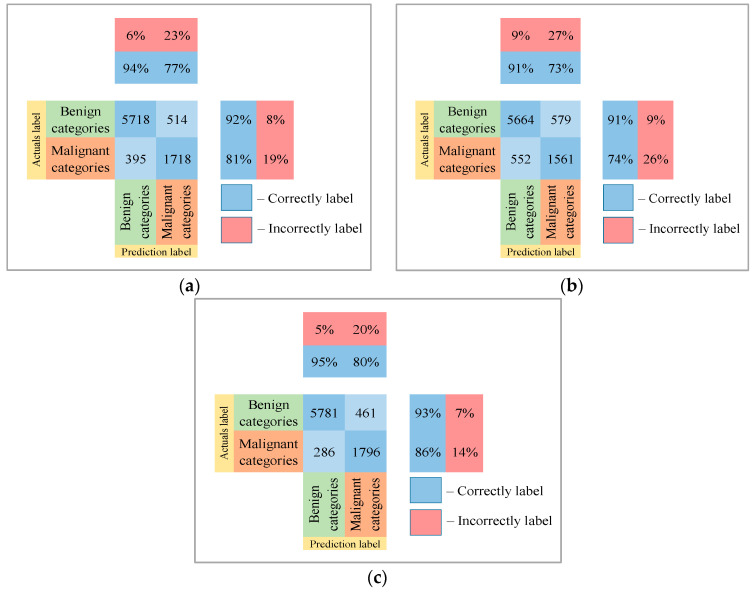
The confusion matrix of the test results of the proposed multimodal neural network system based on CNN is divided into two groups: (**a**) AlexNet; (**b**) SqueezeNet; (**c**) ResNet-101.

Following the analysis of the confusion matrices in Figure 13, Figure 14 and Figure 15, it can be concluded that the most frequently erroneous prediction results concern the different categories of malignant skin neoplasms (see percentages at the top of the columns). As summarized in Figure 16, part of these errors are benign lesions predicted as malignant (i.e. false positives). In addition, the malignant categories of “basal cell carcinoma” and “melanoma” are often predicted as pigmented neoplasms of benign categories. Based on the lines of the confusion matrices in Figure 16, malignant pigmented neoplasms are falsely recognized as benign in an average of 19.6% of cases.

The $\chi^{2}$ McNemar statistic was calculated as follows:

(13) $$\chi^{2}=\frac{{\left(b-c\right)}^{2}}{b+c}$$

where b is the value when the proposed multimodal system incorrectly predicted the images and the results of the original CNN were correct; c is the value when the results of the original CNN were incorrect and the results of the multimodal system were correct.

**Figure 17:**
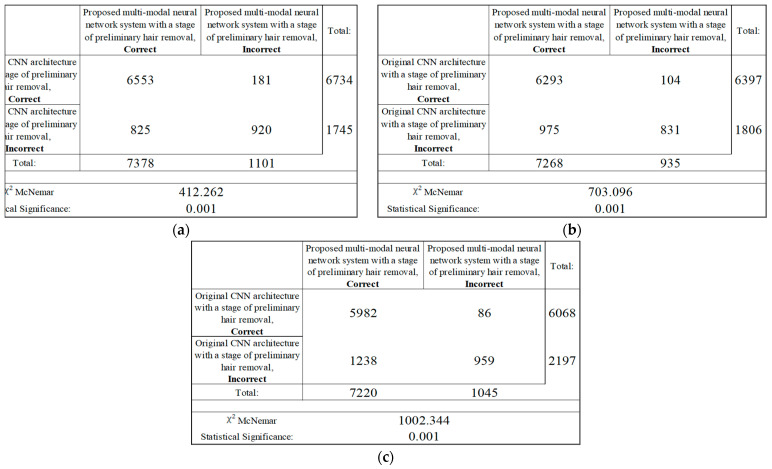
Classification table neural network systems for recognizing pigmented skin lesions for analysis McNemar based on CNN: (**a**) AlexNet; (**b**) SqueezeNet; (**c**) ResNet-101.

The results of the analysis of the McNemar test from Figure 17 show that the proposed multimodal neural network system made it possible to correctly recognize pigmented neoplasms in 825–1238 images that were incorrectly classified by the original CNN with a pre-cleaning step for oatmeal structures; in 86–181 the image was misclassified, in contrast to the results of the original CNN with a pre-cleaning step for oat structures. Based on the results of the McNemar test, the proposed multimodal neural network system correctly classifies images of pigmented neoplasms on average, 12% of the time, compared to the original convolutional neural network architectures with a hair pre-cleaning step.

Even though the proposed multimodal neural network system with the stage of preliminary cleaning of hair structures shows higher results in recognition accuracy compared to existing similar systems, as well as compared to visual diagnostic methods for physicians in the field of dermatology, the use of the proposed system as an independent diagnostic tool is impossible due to the presence of a false-negative response in cases of malignant neoplasms. This system can only be used as a high-precision auxiliary tool for physicians and specialists.

**Figure 18:**
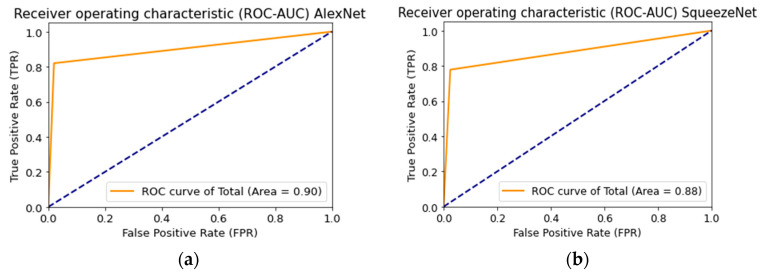

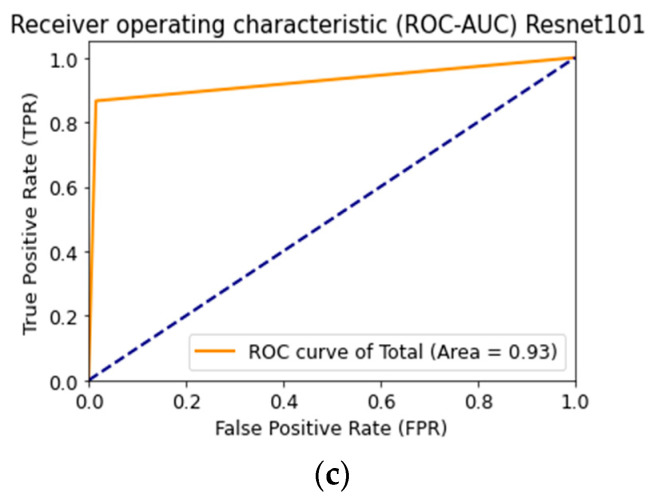
Receiver operative characteristics (ROC) curve when testing a multimodal neural network system for recognizing pigmented lesions and skin based on CNN: (**a**) AlexNet; (**b**) SqueezeNet; (**c**) ResNet-101.

Figure 18 shows the ROC curve when testing a multimodal neural network system to identify pigmented skin lesions based on various CNNs.

AlexNet deep neural network architecture is superior to other architectures in the following ways: it does not require specialized hardware and works well with limited GPU; learning AlexNet is faster than other deeper architectures; more filters are used on each layer; a pooling layer follows each convolutional layer; ReLU is used as the activation function, which is more biological and reduces the likelihood of the gradient disappearing [59]. The listed characteristics substantiate the best result of training a multimodal neural network to recognize pigmented skin lesions based on the AlexNet neural network architecture.

## 4. Discussion

As a result of modeling the proposed multimodal neural network system, the best recognition accuracy was 83.6%. The preliminary cleaning of hair structures and the analysis of heterogeneous data made it possible to significantly exceed the classification accuracy compared to simple neural network architectures to recognize dermoscopic images. In [20] CNN GoogleNet Inception v3 was trained based on dermoscopic images, consisting of nine diagnostically significant categories. The recognition accuracy of CNN GoogleNet Inception v3 was 72.1%, which is 11.46% lower than modeling the multimodal neural network system proposed in this paper; in [21], the authors present CNN ResNet50 training based on benign and malignant pigmented skin lesions. The trained ResNet50 CNN achieved 82.3% accuracy, which is 1.26% lower than the recognition accuracy of the proposed system with the hair pre-cleaning step. The superior recognition accuracy of the multimodal neural network system proposed in this paper compared to the results of pre-trained CNNs is explained by different data processing methods, which, when used together, enter into synergy.

In [60], preliminary hair cleaning is performed using the DullRazor method, and the skin lesion image classification using a neural network classifier. The best result of recognition accuracy was 78.2%. The analysis of heterogeneous data using the proposed multimodal neural network system made it possible to increase the recognition accuracy by 5.4% compared to recognition using a neural network classifier; [61] presents a skin cancer detection system. The preliminary cleaning of dermatoscopic images from hair was performed at the first stage using the DullRazor method. Neural network classification was performed using the K-Nearest Neighbor (KNN). The system’s accuracy was 82.3%, which is 1.3% lower than the recognition accuracy of the proposed multimodal neural network system with the stage of preliminary cleaning of hair structures. The authors of [62] proposed a neural network system for classifying benign and malignant pigmented skin lesions with the stage of preliminary hair removal. This approach made it possible to achieve a classification accuracy of 79.1%, which is 4.5% lower than the recognition accuracy of the proposed multimodal neural network system. Combining and analyzing heterogeneous dermatological data allows the multimodal neural network algorithm to find additional links between images and metadata and improve recognition accuracy compared to the classification accuracy of visual data only by neural network algorithms.

A comparison of the recognition accuracy of various multimodal neural network systems for recognizing pigmented lesions and skin with the proposed system is presented in Table 6.

In [34], the authors solved two problems for neural network classification of pigmented skin lesions. The modeling was carried out based on the open archive ISIC 2019, which is currently the most suitable for research in this area since it contains the largest amount of visual and statistical data. The authors selected 25,331 dermatoscopic images for modeling, divided into eight diagnostically significant categories. The authors used various CNNs to classify dermatoscopic images for the first task. For the second task, statistical metadata about patients was also used along with the photos. The multimodal neural network system for the second task consisted of CNN for dermatoscopic imaging and a dense neural network for metadata. In the first step, the authors trained CNN only on visual multivariate data, then fixed the CNN weights and connected a neural network with metadata. The core architecture of CNN was a pre-trained EfficientNets consisting of eight different models. Pre-trained SENet154 and ResNext were also used for modeling variability. The images were cropped to the required size 224 × 224 × 3 and augmented as a pre-processing stage. Metadata pre-processing consisted of simple numeric coding. In this case, the missing values were coded as “−5”. Most of the training was done on an NVIDIA GTX 1080TI graphics card. The use of metadata has improved the accuracy by 1–2%. At the same time, the increase was observed mainly on smaller models. On the test set, the accuracy of the neural network recognition system in the first task was 63.4%. For the second task using metadata, the accuracy on the test set was 63.4%. At the same time, the most optimal results were 72.5 ± 1.7 and 74.2 ± 1.1 for the first and second tasks, respectively.

Identical conditions for modeling, hardware resources, image base, and many diagnostic categories used make it possible to compare the results obtained with the proposed multimodal neural network system with the stage of preliminary hair removal with the results from work. The recognition accuracy of the proposed multimodal system with the stage of preliminary hair removal on the test set was 83.6%, which is about 20.2% higher than the results of testing the system from [34]. The main difference between the multimodal neural network system proposed in the work is the use of the hair removal method at the stage of preliminary processing of visual data, which significantly increased the accuracy.

In [35], a multimodal convolutional neural network (IM-CNN) is presented, a model for the multiclass classification of dermatoscopic images and patient metadata as input for diagnosing pigmented skin lesions. The modeling was carried out on the open dataset HAM10,000 (“Human versus machine with 10,000 training images”), part of the ISIC Melanoma Project open database, and consists of seven diagnostic categories. This set includes statistical metadata about patients such as age, gender, location of pigmented lesions, and diagnosis. The pre-trained DenseNet and ResNet architectures were used as CNNs to classify dermatoscopic images. The best test result for the proposed model was 72% recognition accuracy. That is about 11.6% lower than the proposed multimodal system with a stage of preliminary hair removal. The main differences in the operation of the proposed multimodal system for the recognition of pigmented lesions of the skin are, firstly, the stage of preliminary hair removal, and, secondly, the use of a larger number of diagnostically significant recognition classes and a more substantial amount of data for training. These distinctive features made it possible to improve the visual quality of diagnostically significant signs on dermatoscopic images due to the removal of hair structures and improve the correctness and balance of the training of the neural network system.

The authors of [36] presented a method combining visual data and patient metadata to improve the efficiency of automatic diagnosis of pigmented skin lesions. The modeling was carried out on the ISIC Melanoma Project database, which consisted of 2917 dermatoscopic images of five classes (nevi, melanoma, basal cell carcinoma, squamous cell carcinoma, pigmented benign keratoses). For image recognition, a modified CNN architecture, ResNet-50, was used. Simulation results have shown that the combination of dermatoscopic images and metadata can improve the accuracy of the classification of skin lesions. The best average recognition accuracy (mAP) using metadata on the test set was 72.9%. This result is 10.7% lower than the recognition accuracy of the proposed multimodal system for recognizing pigmented skin lesions with a stage of preliminary removal of hair structures. A small variation in the database of dermatoscopic examples for training in [36] can significantly affect the reliability of the neural network classification system.

In [38] proposed two methods for classifying pigmented skin lesions. The first method was to use CNN to recognize dermatoscopic images. The authors selected 1000 images from the International Skin Imaging Collaboration (ISIC) archive, divided into two categories (benign and melanoma). The result of recognition accuracy in two categories on the basis for validation was 82.2%. The second method used 600 images from the ISIC archive and patient metadata. Metadata has been added to the dermatoscopic image pixel matrix in each RGB layer at the bottom. After repeatedly adding metadata, a colored bar appeared on the images. The accuracy of CNN recognition and the metadata on the validation set was 79.0%, which is 4.6% lower than the recognition accuracy of the proposed multimodal neural network system. Although adding metadata directly to the image matrix allowed the authors from [38] to improve the classification accuracy, using a separate full-fledged classifier for statistical data is a more rational solution. Convolutional layers in CNN highlight such features on dermatoscopic images as contour, color, size. The metadata added to the pixel matrix of each dermatoscopic image does not require feature extraction.

The main limitation in using the proposed multimodal neural network system for recognizing pigmented lesions in the skin is that specialists can only use the system as an additional diagnostic tool. The proposed system is not a medical device and cannot independently diagnose patients. Since the major dermatoscopic training databases are biased towards benign image classifications, misclassification is possible. The use of augmentation based on affine transformations makes it possible to minimize this factor but not completely exclude it.

A promising direction for further research is constructing more complex multimodal systems for neural network classification of pigmented skin neoplasms. The use of segmentation and preliminary cleaning of the hair’s visual data will help highlight the contour of the pigmented skin lesion. Distortion of the shapes of the skin neoplasm is an important diagnostic sign that may indicate the malignancy of this lesion.

## 5. Conclusions

The article presents a multimodal neural network system for recognizing pigmented skin lesions with a stage of preliminary cleaning from hair structures. The fusion of dissimilar data made it possible to increase the recognition accuracy by 4.93–6.28%, depending on the CNN architecture. The best recognition accuracy for 10 diagnostically significant categories was 83.56% when using the AlexNet pre-trained CNN architecture. At the same time, the best indicator of improving the accuracy was obtained using the pre-trained ResNet-101 architecture and amounted to 6.28%. The use of the stage of preliminary processing of visual data made it possible to prepare dermatoscopic images for further analysis and improve the quality of diagnostically important visual information. At the same time, the fusion of patient statistics and visual data made it possible to find additional links between dermatoscopic images and the results of medical diagnostics, which significantly increased the accuracy of the classification of neural networks.

Creating systems for automatically recognizing the state of pigmented lesions of patients’ skin can be a good incentive for cognitive medical monitoring systems. This can reduce the consumption of financial and labor resources involved in the medical industry. At the same time, the creation of mobile monitoring systems to monitor potentially dangerous skin neoplasms will automatically receive feedback on the condition of patients.

## Figures and Tables

**Table 1:** A coding table for patient gender metadata.

Patient Gender (Sex)	One-Hot Code	
male	0	1
female	1	0

**Table 2:** A coding table for localization of pigmented lesion on the patient body.

Localization of Pigmented Lesion on the Patient Body (Anatomloc)	One-Hot Code							
anterior torso	1	0	0	0	0	0	0	0
head/neck	0	1	0	0	0	0	0	0
lateral torso	0	0	1	0	0	0	0	0
lower extremity	0	0	0	1	0	0	0	0
oral/genital	0	0	0	0	1	0	0	0
palms/soles	0	0	0	0	0	1	0	0
posterior torso	0	0	0	0	0	0	1	0
upper extremity	0	0	0	0	0	0	0	1

**Table 3:** A coding table for patient age metadata.

The Age of the Patient (Age)	One-Hot Code																	
0	1	0	0	0	0	0	0	0	0	0	0	0	0	0	0	0	0	0
5	0	1	0	0	0	0	0	0	0	0	0	0	0	0	0	0	0	0
10	0	0	1	0	0	0	0	0	0	0	0	0	0	0	0	0	0	0
15	0	0	0	1	0	0	0	0	0	0	0	0	0	0	0	0	0	0
20	0	0	0	0	1	0	0	0	0	0	0	0	0	0	0	0	0	0
25	0	0	0	0	0	1	0	0	0	0	0	0	0	0	0	0	0	0
30	0	0	0	0	0	0	1	0	0	0	0	0	0	0	0	0	0	0
35	0	0	0	0	0	0	0	1	0	0	0	0	0	0	0	0	0	0
40	0	0	0	0	0	0	0	0	1	0	0	0	0	0	0	0	0	0
45	0	0	0	0	0	0	0	0	0	1	0	0	0	0	0	0	0	0
50	0	0	0	0	0	0	0	0	0	0	1	0	0	0	0	0	0	0
55	0	0	0	0	0	0	0	0	0	0	0	1	0	0	0	0	0	0
60	0	0	0	0	0	0	0	0	0	0	0	0	1	0	0	0	0	0
65	0	0	0	0	0	0	0	0	0	0	0	0	0	1	0	0	0	0
70	0	0	0	0	0	0	0	0	0	0	0	0	0	0	1	0	0	0
75	0	0	0	0	0	0	0	0	0	0	0	0	0	0	0	1	0	0
80	0	0	0	0	0	0	0	0	0	0	0	0	0	0	0	0	1	0
85	0	0	0	0	0	0	0	0	0	0	0	0	0	0	0	0	0	1

**Table 4:** Results of modeling a multimodal neural network classification system for dermatoscopic images of pigmented skin lesions. Bold font indicates the best result in each column of the table.

CNN Architecture	Results of Recognition			
	Original CNN Architecture, %	Original CNN Architecture with a Stage of Preliminary Hair Removal, %	Proposed Multimodal Neural Network System with a Stage of Preliminary Hair Removal, %	Different in Recognition Accuracy between Original and Proposed Neural Network Systems, %
AlexNet [54]	**78.63**	**80.81**	**83.56**	4.93
SqueezeNet [55]	71.63	73.76	77.87	6.24
ResNet-101 [56]	76.75	79.92	83.03	**6.28**

**Table 5:** Testing results of the proposed multimodal neural network system to recognize pigmented lesions. Bold font indicates the best result in each column of the table.

CNN Architecture	Recognition Accuracy, %	Loss Function	AUC
AlexNet [54]	**83.56**	**0.47**	0.90
SqueezeNet [55]	77.87	0.67	0.88
ResNet-101 [56]	83.03	0.66	**0.93**

**Table 6:** Results of recognition accuracy of various multimodal neural network systems for recognizing pigmented lesions and skin.

Multimodal Neural Network Systems for the Classification of Skin Pigmentation Lesions		Accuracy of Detection of Pigmented Skin Lesions, %
Known neural network systems	[34]	63.4
	[35]	72.0
	[36]	72.9
	[38]	79.0
Proposed neural network system		83.6

## Data Availability

A publicly available dataset was analyzed in this study. This data can be found in https://www.isic-archive.com/#!/topWithHeader/wideContentTop/main, accessed on 7 February 2022. Both the data analyzed during the current study and code are available from the corresponding author upon request.

## Appendix A

Appendix A shows the training and testing graphs of the proposed multimodal neural network systems based on various CNN architectures with preliminary cleaning of hair structures.
